# Impact of anticoagulation and vasoactive medication on regained radial artery patency after catheterization: a case–control study

**DOI:** 10.1186/s40001-018-0324-y

**Published:** 2018-05-22

**Authors:** C. Rammos, A. Burghardt, J. Lortz, O. Azizy, R. A. Jánosi, M. Steinmetz, T. Rassaf

**Affiliations:** Department of Cardiology and Vascular Medicine, West German Heart and Vascular Center Essen, Medical Faculty, University Hospital Essen, Hufelandstr. 55, 45147 Essen, Germany

**Keywords:** Alprostadil, Radial artery access, Cardiac catheterization, Radial artery occlusion

## Abstract

**Background:**

Radial artery access is the primary approach for coronary interventions due to higher safety profile in comparison to femoral access. Radial artery occlusion (RAO) is the main complication of transradial catheterization that can lead to severe symptoms and a permanent artery occlusion. The incidence of RAO after transradial access ranges from 5 to 38% and data regarding treatment is scarce. Whether anticoagulation and vasoactive medication provides an additional benefit in recovery of radial artery patency (RAP) after catheterization has not been investigated in detail.

**Aim:**

The objective was to investigate the impact of anticoagulation and vasoactive medication on regained patency after documented RAO following transradial catheterization.

**Patients and methods:**

Overall 2635 patients were screened. 2215 (84%) catheterizations were performed by femoral and 420 (16%) by radial access. In 30 patients RAO was observed. In case of RAO patients were classified in three groups: Anticoagulation, anticoagulation added with alprostadil and controls. Follow-up was conducted after 3 months with ultrasound and clinical examination.

**Results:**

Eight patients received anticoagulation and 11 patients anticoagulation together with alprostadil. Eleven patients served as controls. Recovery of RAP after catheterization was higher following either treatment (79.5%) compared to controls (0%, *p* = 0.006). Subgroup analysis yielded a higher RAP recovery in patients treated with anticoagulation (62.5%) as compared to controls (0%, *p* = 0.002). No effect on regained RAP was found with additional alprostadil therapy (33.3%) compared to anticoagulation therapy (62.5%, *p* = 0.229).

**Conclusion:**

RAO should be treated with anticoagulation to regain patency. Addition of vasoactive medication does not lead to further beneficial effects. Further research is needed regarding preventive and therapeutic strategies following RAO.

## Background

Transradial access (TRA) is the gold standard for cardiac catheterization with a lower major vascular event rate as compared to the transfemoral approach (TFA) and is recommended by current guidelines [1–3]. The major vascular complication of TRA, the permanent radial artery occlusion (RAO), is frequent and incidence is reported with up to 71% when not using heparin during intervention [4, 5]. Nitroglycerin and verapamil are given during interventions to avoid spasms of the radial artery with good success. To reduce RAO following TRA, devices were developed to optimize post-procedural haemostasis [6, 7]. RAO incidence still ranges between 5 and 38% in several studies [2, 8, 9]. This surprising wide range is presumably generated by the fact, that radial occlusion is asymptomatic in most patients because of the dual blood supply of the hand and that ultrasound control is not a standard examination after catheterization [2, 8, 10]. In case of occlusion, no further radial access of the punctured artery is possible and the radial artery can not be used as a graft for aorto-coronary bypass [11, 12].

Weighing the in most cases asymptomatic or mild clinical course of radial occlusion, an interventional recanalization of the radial artery seems exaggerated and is reserved for profound hand ischemia [13]. Success on radial patency by medical therapy with anticoagulation is reported with 56–87% compared to observation alone [8, 14]. In case of RAO, studies have shown a significant effect of low molecular weight heparin (LMWH) on the RAP rate [8, 9]. Especially for patients with contraindication for oral anticoagulation or severe symptoms, an additive therapy with LMWH is desirable to raise the radial patency rate.

In peripheral arterial disease, vasoactive substances are indicated in critical limb ischemia in case of unsuccessful intervention in case of absent vessels for intervention or in acute arterial occlusions [15, 16]. The prostaglandine E1 analog on alprostadil is an approved and well-tolerated therapy for these indications and has the effect of vasodilatation and collateral vessel formation [17]. Since radial artery occlusion and critical limb ischemia (CLI) share a common pathophysiologic path on first sight, we aimed to investigate the impact of anticoagulation and additional vasoactive substances for recanalization and patency following occlusion of the radial artery.

## Methods

**Fig. 1:**
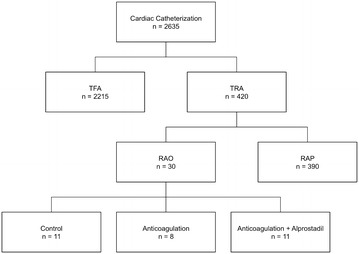
Flowchart of the study. *TFA* transfemoral access, *TRA* transradial access, *RAO* radial artery occlusion, *RAP* radial artery patency

We screened a total of 2635 patients who underwent cardiac catheterisation in a tertiary referral hospital after informed consent in a 1-year timespan. The study flow chart is shown in Fig. 1. 1744 patients underwent diagnostic angiography, while 891 received coronary interventions. 2215 patients were catheterized through the femoral route and in 420 patients a radial access was chosen.

### Transradial artery catheterization

All patients gave their informed consent to perform the procedure by TRA. Eligible patients for TRA had a normal flow profile and a diameter of the radial artery ≥ 2 mm, as documented by duplex ultrasound. For TRA local anaesthesia with xylocaine 2% was applied in case of an unremarkable Allen test. In all cases a 6F sheath (Terumo, Eschborn, Germany) was placed into the radial artery and 5000 IE Heparin together with 0.2 ml nitroglycerin and 1.5 mg verapamil were administered. After angiography the sheath was removed and a compressive device was applied (TR-band, Terumoband, Terumo, Eschborn, Germany). Due to the 6 French sheaths used in this study the inflated air and the TR-band were removed after 3 h, while achieving hemostasis, which represents a modification of the TR Band removal guidelines. In case of persistent bleeding, the removed air was inflated again and hemostasis was checked again after 1 h.

### Vascular ultrasound measurements

Vascular ultrasound was performed from experienced sonographers with Philips IE 33 (Philips GmbH Market DACH, Hamburg, Germany) equipped with a 12 Hz ultrasound linear probe.

Duplex ultrasound of the whole radial artery from brachial bifurcation till the furthest distal point behind the punction site was performed prior and after each TRA. Furthermore aneurysmal or thrombotic transformations were noted. The length of occlusion was specified with division of the forearm in three parts: distal, middle and whole forearm. Symptoms related to RAO were considered pain, paraesthesia, weakness, and algor.

Follow-up was performed on each patient the day after catheterization. In case of occlusion, the ulnar and interosseous artery were checked to guarantee blood supply of the hand. Symptoms, haematoma or absent pulses were checked and follow-up was conducted after 4 and 12 weeks in case of RAO. At follow-up, each patient received ultrasonic duplex control and clinical examination regarding RAO. Endpoint definition was regained RAP.

### Anticoagulation and vasoactive medication

Anticoagulation was performed with LMWH (weight adapted, twice in a day), rivaroxaban (15 mg twice a day for 21 days, then 20 mg once in a day) or apixaban (5 mg twice a day for 5 days, then 5 mg once in a day). Alprostadil (20 µg) was applied i.v. in 250 ml sodium chloride twice a day for the duration of hospital stay. In case of persistent RAO, anticoagulation was continued for a maximum of 3 months.

### Statistics

Statistical analysis was made with SPSS (IBM SPSS statistics version 24 for MAC). Dichotomous variables are reported as numbers and percentages, continuous variables as mean ± SD. Statistical analysis of dichotomous groups was performed by *χ*^2^ test. Patency probability was estimated with Kaplan–Meyer analysis and tested with log rank test. A *p* value < 0.05 was considered statistically significant.

## Results

From 2635 cardiac catheterization 2215 were conducted by transfemoral access and 420 by radial access. Of the 420 patients with radial access, a RAO was documented in 30 patients by duplexsonography (Fig. 1). Baseline characteristics of these patients are given in Table 1.

**Table 1:** Baseline characteristics of patients with occlusion of radial artery after catheterization

Baseline characteristics of study population	*n* (%, ± SD)
Patients at all	30 (100)
Age (years)	55.33 ± 10.48
Height (cm)	175 ± 5.9
Weight (kg)	86 ± 25.6
BMI (kg/m^2^)	28.23 ± 7.75
Sex	
Men	21 (70)
Women	9 (30)
Follow-up in days	164.17 ± 153.175
Indication for catheterization	
Instable angina	18 (60)
Diagnostic angiography	7 (23.3)
NSTEMI	4 (13.3)
STEMI	1 (3.3)
Procedure time (min)	25.3 ± 15.45
Antiplatelet therapy	
Aspirin	10 (33.3)
Aspirin and clopidogrel	12 (40%)
None	8 (26.7%)
Medical therapy of RAO	
LMWH	10 (52.6)
Rivaroxaban	6 (31.6)
Apixaban	1 (5.3)
Single alprostadil	2 (10.5)
Duration of alprostadil therapy (days)	7.91 ± 3.44
Side of occlusion	
Left radial artery	20 (66.7)
Right radial artery	10 (33.3)
Length of occlusion	
Distal forearm	13 (43.3)
Distal and middle forearm	6 (20)
Whole forearm	11 (36.7)
No symptoms	14 (46.7)
Symptoms	16 (53.3)
Pain	16 (100)
Paresthesia	2 (12.5)
Weakness	1 (6.25)

Mean age was 55.33 ± 10.48 years (male 70%, female 30%). Indication for catheterization was unstable angina in 18 patients (60%) followed by seven patients (23.3%) with diagnostic catheterization due to valvular heart disease. Four patients (13.3%) had non-ST-elevation myocardial infarction and one patient (3.3%) ST-segment elevation myocardial infarction. Pain of the forearm was evident in all symptomatic cases and occurred with fading of local anaesthesia. Weakness occurred in one patient and paraesthesia in two patients.

From the 30 patients with RAO 11 patients did not receive drug therapy because RAO was an accidental diagnosis and served as controls. 19 patients showed RAO in duplexsonography after intervention and received drug therapy in form of single anticoagulation (with LMWH or NOAC) or anticoagulation added with alprostadil.

As mentioned above, the medical treated group was divided in an anticoagulation group (eight patients) and an anticoagulation plus alprostadil group (11 patients). Overall anticoagulation consisted in ten cases of LMWH, in 6 cases of rivaroxaban and in one case of apixaban in a patient with atrial fibrillation. Because of contraindications for anticoagulation, two of the 11 patients in the alprostadil group (one patient with gastrointestinal bleeding and one with ischemic insult in the last 2 weeks) received single alprostadil therapy for 14 days. Vasoactive therapy was performed for 7.91 ± 3.44 days (range 5–14 days) with 20 µg alprostadil in 250 ml sodium chloride twice a day applied over 2 h.

Follow-up was conducted at 164.17 ± 153.17 days. At the end of follow-up, 22 patients (73.3%) still had RAO while 8 patients (26.7%) showed RAP.

**Fig. 2:**
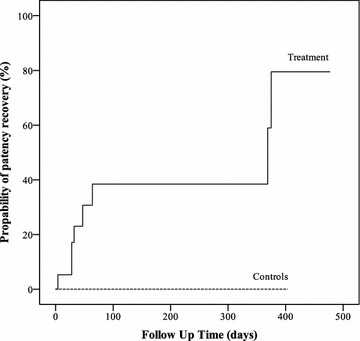
Probability of patency recovery after radial artery occlusion following catheterization in controls and treated patients. A higher patency probability was observed with either anticoagulation or anticoagulation with additional alprostadil than in controls (*p* = 0.006)

Patients with any kind of therapy (*n* = 19) showed higher probability of regained RAP (79.5%) at the end of follow-up as compared to the control group (*n* = 11, 0%, *p* = 0.006, Fig. 2).

**Fig. 3:**
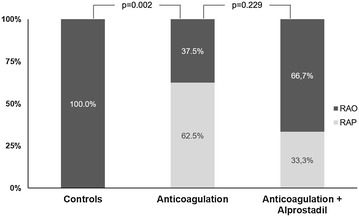
Patency rate after RAO following cardiac catheterization. Comparison of RAO patency in the Controls and in patients treated with anticoagulation, with higher patency in the anticoagulation group (*p* = 0.002). No benefit in RAO patency in patients with anticoagulation and alprostadil treatment as compared to anticoagulation (*p* = 0.229); *RAO* radial artery occlusion, *RAP* radial artery patency

Anticoagulation serves as the gold standard in thrombotic arterial occlusions. We thus first compared control subjects with subjects receiving anticoagulation. The positive effect of anticoagulation on patency was confirmed by the higher patency in the patients who received anticoagulation compared to controls (62.5% vs. 0%, *p* = 0.002, Fig. 3).

We then sought to compare the best medical treatment with the additional impact of vasoactive medication. No further effect on the patency was found in the alprostadil therapy group as compared to the anticoagulation group (33.3% vs. 62.5%, *p* = 0.229, Fig. 3).

## Discussion

TRA has grown to become the preferred access in cardiac catheterization since first undertaken in the 90 s of the last century. It leads to less bleeding complications, higher patient comfort with earlier mobilization and therewith, lower procedure-related costs. Especially in patients with co-morbidities like severe peripheral disease or aortic diseases it is a feasible approach for cardiac catheterization.

RAO is main complication of transradial catheterization that can lead to permanent occlusion of the radial artery. Clinical relevance is not obvious at first sight because cases of critical hand ischemia are rare. RAO has an incidence up to 38% [18, 19]. These factors accompany the necessity for RAO prevention, duplexsonography control before discharge and therapeutic treatment in case of RAO.

Based on the pathophysiologic principle of RAO with endothelial injury involving thrombotic occlusions and the positive peri-procedural effect of heparin, different studies showed a positive effect of heparin on radial artery recanalization [5, 8, 14, 18, 20–23]. Our study confirms the positive effect of anticoagulation on RAP with higher patency probability of 79.5% and higher patency rate. First studies showed a preventive effect of peri-interventional heparin application of a minimum of 5000 IE unfractionated heparin [10, 18]. Predictors for RAO like small radial diameter, sheath size, gender, long procedure time and co-morbidities like diabetes are controversially discussed. The relation between increasing heparin dose and RAO prevention was previously described [5, 10, 18]. A relation between RAO and end-procedural activated clotting time was shown [5]. Non-occlusive hemostasis with the TR Band seems to prevent RAO. With the use of heparin while intervention and the TR band as the compressive device RAO incidence in our cohort was comparable to other studies with 7.14% [4, 8, 9, 20].

We investigated the effect of vasoactive medication on RAP. Since no alternative therapeutic approaches exist for patients with contraindications for anticoagulation, our study provides important findings. We added alprostadil to anticoagulation, but without additional therapeutic effect compared to anticoagulation alone. Regained RAP was even lower in the anticoagulation and alprostadil group (33.3%) than in the anticoagulation only group (62.5%, Fig. 3), so a negative effect can be assumed on first sight; however, this is more a statistical effect due to small sample size.

CLI is the end stage of peripheral artery disease PAD disease and a slow process over years that leads to rest pain, tissue loss and necrosis [24]. The mechanisms of PGE1 in CLI are not only based on active vasodilation, but are considered to be due to inhibition of expression of adhesion molecules, release of inflammatory cytokines and generation and release of growth factors, amongst others, which might be missing in RAO [25]. The explanation for the missing therapeutic effect might be due to the not entirely identical pathophysiology of CLI treated with vasoactive medicine and the “missing” critical ischemia in RAO patients, because of the dual blood supply of the hand.

### Limitations

From 420 patients only 30 patients showed RAO. In this small retrospective analysed cohort of 30 patients the ineffectiveness of vasoactive substances in therapy of RAO has to be taken with caution, albeit the significant higher RAP rate in the anticoagulation group corroborates the results of other studies. Follow-up time between the therapy group and the control group was very inhomogeneous due to the fact that the control group had not predefined ultrasonographic control. Other RAO predictors like gender, diameter of the radial artery, procedure and compression time or antegrade flow during homeostasis were not included in the analysis of this study due to small sample size. Additional studies should investigate the optimal anticoagulation regimen, whether LMWH or NOAC should be used for treating RAO. Moreover, the optimal duration of therapy has to be elucidated. For future investigations, prospective and potentially randomized trials are needed, to determine the impact of RAO predictors and best medical treatment.

## Conclusions

This is the first study to investigate the effects of anticoagulation and additional vasoactive medication on regained patency after RAO. Our study confirms the effectiveness of anticoagulation in therapy of RAO, while the addition of vasoactive medication does not seem to confer to additional benefit. Further research is needed regarding preventive and therapeutic strategies following RAO.

## Abbreviations

CLI: critical limb ischemia
LMWH: low molecular weight heparin
NOAC: noval oral anticoagulation
PAD: peripheral artery disease
RAO: radial artery occlusion
RAP: radial artery patency
TFA: transfemoral access
TRA: transradial access
